# The Fouling of Zirconium(IV) Hydrous Oxide–Polyacrylate Dynamically Formed Membranes during the Nanofiltration of Lactic Acid Solutions

**DOI:** 10.3390/membranes3040415

**Published:** 2013-12-10

**Authors:** Ewa Polom

**Affiliations:** Department of Chemical Engineering, West Pomeranian University of Technology, Szczecin, al. Piastów 42, 71-065 Szczecin, Poland; E-Mail: ewa.polom@zut.edu.pl; Tel.: +48-914-494-472; Fax: +48-914-494-642

**Keywords:** nanofiltration, dynamically formed membranes, lactic acid, fouling, resistance in series model

## Abstract

The results of investigations of flux decline during nanofiltration (NF) of lactic acid solutions using dynamically formed zirconium(IV) hydrous oxide/polyacrylate membranes (Zr(IV)/PAA) under conditions resulting in low and high lactic acid rejection are reported. The experimental permeate flux *versus* time curves were analyzed in the frame of resistance in a series model with the aim of developing the characteristic of resistances. Analysis of experimental data and results of calculations showed that the reduction of fouling effects in the investigated system could be achieved due to appropriate hydrodynamic process conditions and regular rinsing with deionized water.

## 1. Introduction

The development of environmentally friendly technologies must include the application of membrane processes into conventional ones, which allows us to save energy and substrates. Dynamically formed membranes are regarded as the most interesting separation medium in this area. The morphological characteristic of Zr(IV)/PAA membrane has been presented in previous works [1,2]. The dynamically formed membranes have some interesting advantages: high mechanical and chemical resistance, long life time, high thermal stability, possibility of *in situ* membrane replacement and high permeability corresponding to high rejection. They have only a few disadvantages: relatively high construction cost and considerable weight as well as low rate of compaction of membrane modules [3,4].

These kinds of membranes can work in conditions resulting in low as well as in high solute rejection. Hence, they can be used for concentration and purification processes like lactic acid recovery from waste lactose obtained from whey.

There are three main subprocesses in the fermentation of sugars: feedstock preparation, sugar conversion, product recovery and concentration, which correspond to subsequent steps in the whole hybrid process: upstream processing, bioreactor and downstream processing. In upstream processing, raw materials are prepared before being placed in a bioreactor. The membranes are used for preconcentration, purification and sterilization.

The distinctive feature of bioprocess is the necessity of separating precisely the desired products from multicomponent and usually much diluted systems. Thus, it is a separation that is of fundamental importance for such technology. Both membranes and conventional units can be used for the purposes of upstream and down stream processing. Bioreactors may also be employed.

In the bioreactor, membranes are mainly used for immobilization of microorganisms in reaction space, which is important from economical point of view. In downstream processing, the final product is in multicomponent and diluted fermentation broth. Bioreactors coupled with ultrafiltration (UF) or NF units permit continuous removal of lactic acid from the fermentation broth, recovery of substrate and preliminary purification of a product. Lactose preconcentration, obtained from fermentation broth can be realized using such membrane processes as reverse osmosis (RO) or nanofiltration (NF). Final product concentration to the commercial level provide RO, NF and also conventional units such as evaporation. The membrane approach for the production of lactic acid from waste lactose is more or less hypothetical. In practice, some conventional units (evaporation, neutralization, filtration, distillation) may be connected with the membranes’ processes or partially replaced by them [3].

In previous papers, NF studies using high pressure Zr(IV)/PAA dynamically formed membranes and lactic acid solutions with employing a statistical experimental design were reported [2,3]. The aim of the research in previous works was the attainment of a range of optimum conditions for nanofiltration resulting in low and high lactic acid rejections. The Zr(IV)/PAA membrane can be used for removal of lactic acid from the fermentation broth (low value of lactic acid rejection) and preconcentration of lactic acid solution (high value of lactic acid rejection).

The polynomial equation that is developed as a result of an application of the design procedure for characterization of the nanofiltration process in lactic acid solutions has provided an evaluation of level of operating parameters, resulting in minimum and maximum lactic acid rejection. 

This paper focuses on the description of flux decline in NF for operating parameters providing low and high lactic acid rejection with use of resistance in a series model.

## 2. Experimental

### 2.1. Zr(IV)/PAA Membrane Properties

High-pressure Zr(IV)/PAA membranes were used in research on nanofiltration process of lactic acid model solutions. They were formed on a permanent titanium dioxide microfiltration membrane support with internal diameter, 0.016 m and area, 0.029 m^2^, produced by Du Pont Separation Systems (USA), in a two step dynamic process [1,2]. Replacement of Zr(IV)/PAA membrane is possible by a three-step rinsing process.

### 2.2. Cross-Flow Filtration Unit

**Figure 1:**
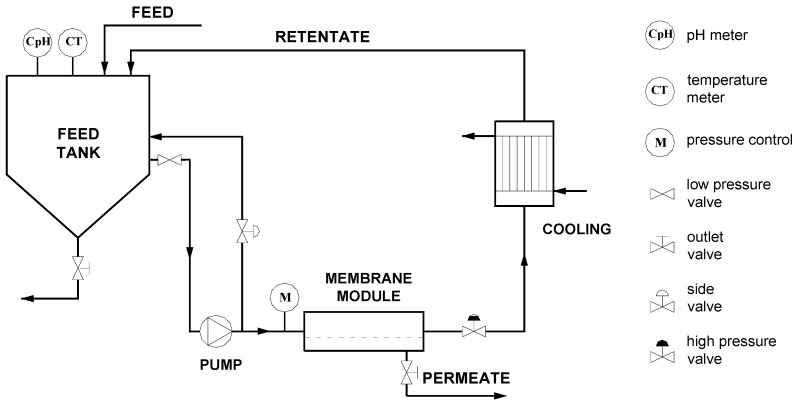
Scheme of experimental set-up.

The research on nanofiltration process of lactic acid solutions was performed with use of high pressure membrane installation equipped with tubular module and zirconium(IV) hydrous oxide dynamically formed membrane. The side valve gave the possibility of velocity regulation at a constant pressure. The scheme of the experimental set up is presented in Figure 1.

The feed tank (40 liter of volume) was coupled with the membrane module. The temperature was regulated with a heat exchanger on the retentate circulation loop and was maintained at the level of 40 °C during the time of measurements. 

During the experiments lactic acid solutions were pumped from the feed tank to the one pipe membrane module in a cross-flow filtration mode and then both permeate and retentate were recirculated back to the feed tank. After each measurement, the fouled membrane was rinsed with deionized water in 3 h periods at an open permeate outlet valve. This way of research set-up gave possibility to estimate an impact of operating parameters on permeate flux reduction.

Concentration of lactic acid was measured by HPLC method with 440 Absorbance Detector supplied by Waters Associates. Samples of retentate and permeate were collected for analysis in 30 min periods during first 3 h and then each hour of the experimental cycle.

### 2.3. Operating Conditions of Nanofiltration Experiments

NF experiments were performed at constant temperature of 40 °C for operating parameters resulting in low and high lactic acid rejection. The operating parameters for analysis of concentration polarization and fouling phenomena evaluated earlier using statistical design method are presented in Table 1 [2,4].

**Table 1:** Values of operating parameters chosen for flux decline analysis [2,4].

Lactic acid rejection	Parameters			
*r*_min_	*u*, m/s	∆*p*, MPa	pH	*c_LA_*, mol/dm^3^
0.02	2.6	1.4	4.0	0.02
0.09	1.0	1.4	4.0	1.00
*r*_max_	*u*, m/s	∆*p*, MPa	pH	*c_LA_*, mol/dm^3^
0.82	1.0	5.5	8.0	0.02
0.58	2.6	5.5	8.0	1.00

The investigated nanofiltration system provides low lactic acid rejection for both low (*c_LA_* = 0.02 mol/dm^3^) and high (*c_LA_* = 1.0 mol/dm^3^) lactic acid concentration in the feed solution at different velocity values 2.6 m/s and 1.0 m/s. The high lactic acid rejection value have been obtained for lactic acid concentration increased from 0.02 to 1.0 mol/dm^3^ at pH 8.0 and cross-flow velocity decreased from 2.6 to 1.0 m/s at transmembrane pressure 5.5 MPa.

## 3. Results and Discussion

### 3.1. Flux Decline Analysis

**Figure 2:**
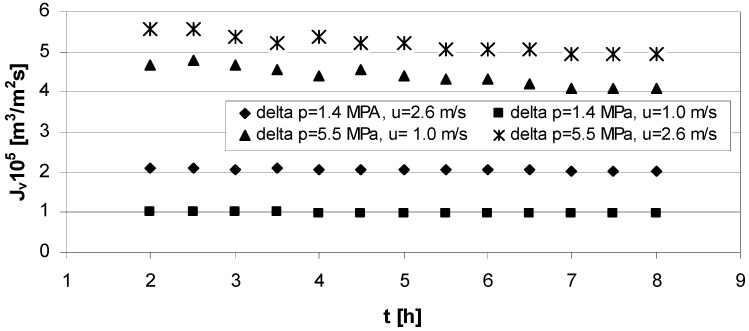
Permeate flux through clean membrane *vs.* time for: series 1, 2—transmembrane pressure ∆*p* = 1.4 MPa, and cross flow velocity *u* = 2.6 m/s and *u* = 1.0 m/s respectively and series 3, 4—transmembrane pressure ∆*p* = 5.5 MPa, cross flow velocity *u* = 1.0 m/s and *u* = 2.6 m/s respectively.

**Figure 3:**
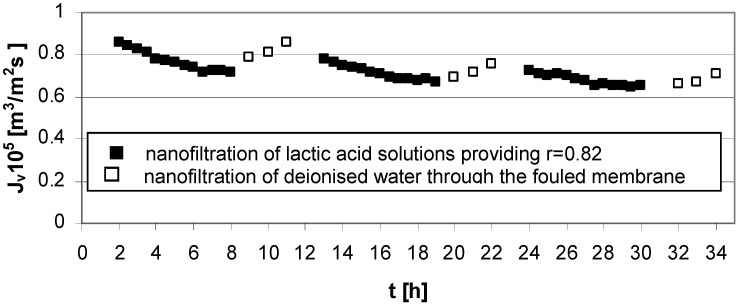
Permeate flux decline *vs.* time for operating parameters providing high lactic acid rejection, *r* = 0.82 (Table 1); series 1, 2, 3—nanofiltration of lactic acid solutions; series 1w, 2w, 3w—nanofiltration of water (“w” is the DI symbol for each series); concentration of lactic acid *c_LA_* = 0.02 mol/dm^3^, pH = 8.0, transmembrane pressure ∆*p* = 5.5 MPa, cross flow velocity *u* = 1.0 m/s.

**Figure 4:**
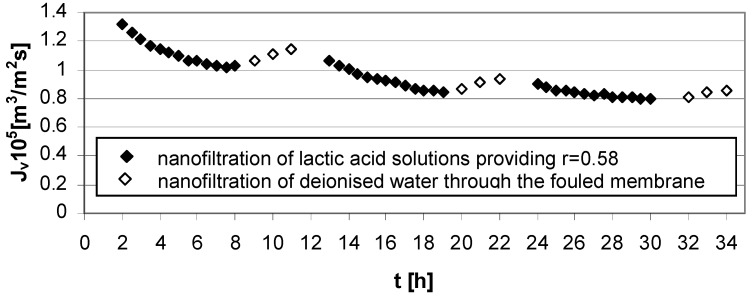
Permeate flux decline *vs.* time for operating parameters providing high lactic acid rejection, *r* = 0.58 (Table 1); series 1, 2, 3—nanofiltration of lactic acid solutions; series 1w, 2w, 3w—nanofiltration of water; concentration of lactic acid *c_LA_* = 1.0 mol/dm^3^, pH = 8.0, transmembrane pressure ∆*p* = 5.5 MPa, cross flow velocity *u* = 2.6 m/s.

**Figure 5:**
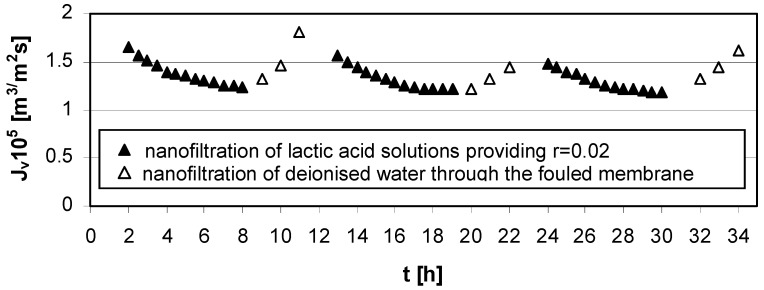
Permeate flux decline *vs.* time for operating parameters providing low lactic acid rejection, *r* = 0.02 (Table 1); series 1, 2, 3—nanofiltration of lactic acid solutions; series 1w, 2w, 3w—nanofiltration of deionized water; concentration of lactic acid *c_LA_* = 0.02 mol/dm^3^, pH = 4.0, transmembrane pressure ∆*p* = 1.4 MPa, cross flow velocity *u* = 2.6 m/s.

**Figure 6:**
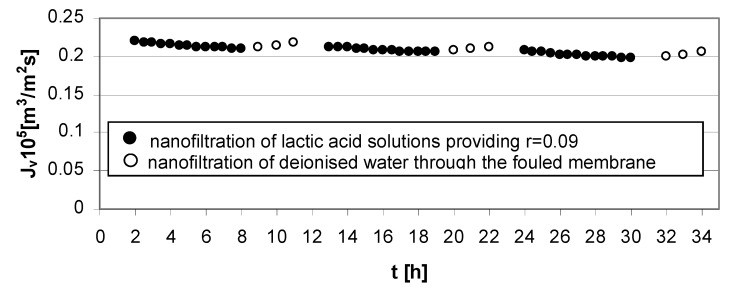
Permeate flux decline *vs.* time for operating parameters providing low lactic acid rejection, *r* = 0.09 (Table 1); series 1, 2, 3—nanofiltration of lactic acid solutions; series 1w, 2w, 3w—nanofiltration of deionized water; concentration of lactic acid *c_LA_* = 1.0 mol/dm^3^, pH = 4.0, transmembrane pressure ∆*p* = 1.4 MPa, cross flow velocity *u* = 1.0 m/s.

Three types of flux measurements were performed: NF of water through clean membrane; NF of lactic acid solutions; NF of water through fouled membrane. The obtained results are presented in Figure 2, Figure 3, Figure 4, Figure 5 and Figure 6.

The experimental results of deionized water filtration process at the different transmembrane pressure and cross-flow velocity is presented in Figure 2 as a function of permeate flux *versus* time. 

Characteristic of flux decline during the experiments of nanofiltration of lactic acid solutions changed due to the operating parameters.

The results presented in Figure 3 indicate that, after experiments at low acid concentration (*c_LA_* = 0.02 mol/dm^3^), high transmembrane pressure (∆*p* = 5.5 MPa), and low cross flow velocity (*u* = 1.0 m/s) cleaning the Zr(IV)/PAA membrane with deionized water is rather effective. 

The results of the experiment presented in Figure 4 show a connection between high acid concentration (*c_LA_* = 1.0 mol/dm^3^), high transmembrane pressure (∆*p* = 5.5 MPa) in NF process conditions and Zr(IV)/PAA membrane usage. Strong fouling effects were generated and the implemented method of rinsing with deionized water was not particularly effective. 

At conditions of low concentration of lactic acid (*c_LA_* = 0.02 mol/dm^3^), low transmembrane pressure (∆*p* = 1.4 MPa), and high cross flow velocity (*u* = 2.6 m/s), the method of rinsing nanofiltration Zr(IV)/PAA membrane with deionized water implemented after each experimental series was effective in reducing fouling phenomena. 

The mass transfer resistance is connected with the solution feed properties. The high lactic acid concentration in the feed solution generates the reduction of permeate flux at high (Figure 4) and low transmembrane pressures (Figure 6).

The strong impact on efficiency decline of the lactic acid nanofiltration process is observed for process parameters taken from upper limit, such as high value of transmembrane pressure, ∆*p* = 5.5 MPa, pH = 8.0 and lactic acid concentration, *c_LA_* = 1.0 mol/dm^3^ (Figure 4). Configured in such conditions, the operational parameters of the experiment cause problems with effectiveness of water cleaning in short 3 h terms.

The effect of pH on both selectivity as well as the effectiveness of the nanofiltration process is connected with characteristic behavior of Zr(IV)/PAA membranes. The electrolyte rejection increases with increasing of pH in the feed solutions due to an increase in membrane charge (Figure 3 and Figure 4) [5].

For the presented results, the impact of both transmembrane pressure and cross-flow velocity could not be ignored. Increasing cross-flow velocity reduces the negative consequences of concentration polarization and fouling (Figure 5). 

### 3.2. Fouling Resistances

The experimental data concerning flux decline *versus* time allow us to estimate the main mass transport resistances caused by fouling phenomena: reversible fouling resistance *R_rf_* and irreversible fouling resistance *R_if_* [6].

According to the resistance in the series model, permeate fluxes during nanofiltration processes of lactic acid solutions, *J_vk_* and deionized water through fouled membrane, *J_wk_* can be expressed by Equations (1)–(4):

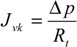
(1)

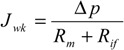
(2)
where:

*R_t_* = *R_m_* + *R_pf_* (3)

*R_pf_* = *R_rf_* + *R_if_* (4)

*R_t_* is the value of total membrane fouling, and;

*R_pf_* is the value of resistance caused by concentration polarization and fouling.

The active membrane layer resistance, *R_m_*, was determined using experimental data obtained during the nanofiltration of deionized water through clean membrane for investigated values of cross-flow velocity, *u* and transmembrane pressure, Δ*p* (Table 1).

The value of *R_m_* was obtained according to the results presented in Figure 2 and calculated from Equation (1) where *R_m_* was *R_t_* for clean membrane. 

The experimental results presented in Figure 3, Figure 4, Figure 5 and Figure 6 were the data for determined *R_t_* and *R_pf_* values with the resistance in series model usage according to Equations (1)–(4). The values of characteristic resistances calculated using Equations (1)–(4) are summarized in Table 2. 

The active membrane layer resistance, *R_m_* and values of concentration polarization and fouling, *R_pf_* depends strongly on dynamic process conditions, especially on cross-flow velocity. The results collected in Table 2 show that the lowest values of active membrane layer resistance, *R_m_*, were obtained for cross-flow velocity *u* = 2.6 m/s as well as values of concentration polarization and fouling. Experimental conditions with low lactic acid concentration in feed solution, *c_LA_* = 0.02 mol/dm^3^, low cross-flow velocity *u* = 1.0 m/s and high trans membrane pressure, ∆*p* = 5.5. MPa with pH = 8.0 generate the highest value of fouling in range of 5.06 × 10^5^–6.40 × 10^5^.

The lowest value of irreversible fouling resistance was obtained for low lactic acid concentration in feed solution, *c_LA_* = 0.02 mol/dm^3^, pH = 4.0, low transmembrane pressure, ∆*p* = 1.4 MPa and high cross-flow velocity, *u* = 2.6 m/s.

**Table 2:** Experimental results of permeate fluxes and calculated values of fouling resistances for nanofiltration process of lactic acid solutions with Zr(IV)/PAA dynamically formed membranes.

Rejection of lactic acid	*r* = 0.02			*r* = 0.09			*r* = 0.58			*r* = 0.82		
Series number	1	2	3	1	2	3	1	2	3	1	2	3
*t*, h	8.0	8.0	8.0	8.0	8.0	8.0	8.0	8.0	8.0	8.0	8.0	8.0
*J_vk_* × 10^5^, m^3^/m^2^ s	1.24	1.21	1.19	0.21	0.20	0.19	1.03	0.86	0.80	0.72	0.67	0.65
*R_t_* × 10^−5^, MPa m^2^ s/m^3^	1.13	1.16	1.18	6.67	6.83	7.07	5.34	6.40	6.88	7.64	8.20	8.45
*R_m_* × 10^−5^, MPa m^2^ s/m^3^	0.69	0.69	0.69	1.43	1.43	1.43	1.12	1.12	1.12	1.34	1.34	1.34
*R_pf_* × 10^−5^, MPa m^2^ s/m^3^	0.44	0.47	0.49	5.24	5.40	5.64	4.22	5.28	5.76	6.30	6.86	7.11
*J_wk_* × 10^5^, m^3^/m^2^ s	1.81	1.44	1.61	0.22	0.21	0.20	1.15	0.94	0.86	0.86	0.75	0.71
*R_if_* × 10^−5^, MPa m^2^ s/m^3^	0.08	0.28	0.18	4.99	5.17	5.40	3.66	4.74	5.28	5.06	5.99	6.40
*R_rf_* × 10^−5^, MPa m^2^ s/m^3^	0.36	0.19	0.31	0.25	0.23	0.24	0.56	0.54	0.48	1.24	0.87	0.71
*R_if_*, % *R_t_*	7	24	15	75	76	77	69	74	77	66	73	76
*R_rf_*, % *R_t_*	32	16	26	4	3	3	10	8	7	16	11	8
*R_m_*, % *R_t_*	61	60	59	21	21	20	21	18	16	18	16	16

The analysis of experimental results of lactic acid solutions during nanofiltration process with Zr(IV)/PAA membranes usage confirm that the hydrodynamic conditions of experiments and properties of feed solution have the strong impact on flux decline and fouling of tested membranes.

The method of cleaning the membrane with deionized water is the effective in case of removing fouling generated in low pressure and high velocity experimental conditions.

## 4. Conclusions

In the long term experimental research on pressure driven membrane processes, the main reason for permeate flux reduction is an accumulation of concentration polarization and fouling effects. In this work, the values of fouling and concentration polarization resistance as well as membrane active layer resistance in nanofiltration process of lactic acid solutions have been determined for chosen operational parameters with the application of resistance in a series model.

The performance of membrane with varying process parameters in terms of extent of flux reduction was evaluated. The active membrane layer membrane resistance, *R_m_*, and values of concentration polarization and fouling, *R_pf_* strongly depended on hydrodynamic nanofiltration conditions.

It was found that the major effect of mass transfer resistance is bound with properties of Zr(IV)/PAA membrane and operational parameters. A major effect on flux reduction and the effectiveness of the cleaning membrane has been observed for high concentration of lactic acid in feed solution *c_LA_* = 1.0 mol/dm^3^, transmembrane pressure, ∆*p* = 5.5 MPa and pH = 8.0.

It was found that the method of rinsing Zr(IV)/PAA membrane with deionized water is effective in case of reduce fouling effects, but the effectiveness of such process depends on the degree of membrane resistance to the fouling. 

The Zr(IV)/PAA membrane may be successfully used in a nanofiltration process of lactic acid solutions if the operational parameters enabling the achievement of satisfactory values of retention coefficient and permeate flux have been properly selected. The selection of the latter values should give the best chance for membrane application in the separation and concentration of lactic acid solutions.
